# Smart Flexible Electronics‐Integrated Wound Dressing for Real‐Time Monitoring and On‐Demand Treatment of Infected Wounds

**DOI:** 10.1002/advs.201902673

**Published:** 2020-01-10

**Authors:** Qian Pang, Dong Lou, Shijian Li, Guangming Wang, Bianbian Qiao, Shurong Dong, Lie Ma, Changyou Gao, Zhaohui Wu

**Affiliations:** ^1^ MOE Key Laboratory of Macromolecular Synthesis and Functionalization Department of Polymer Science and Engineering Zhejiang University Hangzhou 310027 China; ^2^ College of Computer Science and Technology Zhejiang University Hangzhou 310027 China; ^3^ Key Laboratory of Advanced Micro/Nano Electronic Devices and Smart Systems of Zhejiang College of Information Science and Electronic Engineering Zhejiang University Hangzhou 310027 China

**Keywords:** flexible electronics, monitoring, on‐demand therapy, UV‐responsive antibacterial hydrogels, wound infection

## Abstract

As the most frequent wound complication, infection has become a major clinical challenge in wound management. To overcome the “Black Box” status of the wound‐healing process, next‐generation wound dressings with the abilities of real‐time monitoring, diagnosis during early stages, and on‐demand therapy has attracted considerable attention. Here, by combining the emerging development of bioelectronics, a smart flexible electronics‐integrated wound dressing with a double‐layer structure, the upper layer of which is polydimethylsiloxane‐encapsulated flexible electronics integrated with a temperature sensor and ultraviolet (UV) light‐emitting diodes, and the lower layer of which is a UV‐responsive antibacterial hydrogel, is designed. This dressing is expected to provide early infection diagnosis via real‐time wound‐temperature monitoring by the integrated sensor and on‐demand infection treatment by the release of antibiotics from the hydrogel by in situ UV irradiation. The integrated system possesses good flexibility, excellent compatibility, and high monitoring sensitivity and durability. Animal experiment results demonstrate that the integrated system is capable of monitoring wound status in real time, detecting bacterial infection and providing effective treatment on the basis of need. This proof‐of‐concept research holds great promise in developing new strategies to significantly improve wound management and other pathological diagnoses and treatments.

## Graphical abstract

A smart flexible electronics‐integrated wound dressing is designed for real‐time monitoring and on‐demand therapy of infected wounds. It is demonstrated that the integrated wound dressing can diagnose infection during early stages by real‐time wound‐temperature monitoring and provide antibacterial treatment based on the collected data via Bluetooth to achieve a closed‐loop wound healing system.

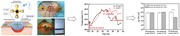

## 1 Introduction

Pathogenic infection as the most frequent complication of chronic wounds has become a global healthcare challenge because of the impairment of the healing process and the risks for amputations and mortality.^1^, ^2^ Currently, dressings in the forms of film, sponge, and hydrogel among others remain the main choice in clinical wound management.^3^, ^4^, ^5^ However, it is difficult to simultaneously obtain the real wound status and meet the dynamic need of a chronic wound under the passive treatment of current wound dressings.^6^, ^7^ The early detection of infection and in‐time treatment on the basis of wound need is of significant importance and should be considered in the next generation of wound dressing. Therefore, a smart wound‐dressing system, which can overcome the “Black‐Box” status of traditional wound management by being integrated with the properties of real‐time monitoring and on‐demand therapies, is greatly required.

With the emergence of flexible electronics, which possess unique properties to enable a natural interaction between electronics and the human body, a wearable healthcare device that integrates multiple sensors and actuators into flexible substrates to achieve conformal contact with skin has provided new ideas for health monitoring, disease diagnosis, and on‐demand treatment, because such a device can collect meaningful physiological data from the human body and diagnose the status in time for dynamic intervention.^8^, ^9^ To date, researchers have developed many forms of wearable devices to monitor body temperature,^10^, ^11^ heart rate,^12^ blood pressure,^13^, ^14^ blood oxygen level,^15^, ^16^ and sweat markers^17^ to evaluate human health. Therefore, with the achievement of a wearable healthcare device, a new generation of smart wound dressing, which consists of sensors capable of detecting physicochemical signals relevant to the wound‐healing process, is emerging to address these problems of current wound dressing. It is reasonable to expect that smart wound dressing can provide real‐time monitoring and point‐of‐care diagnosis, and that physicochemical data can be collected via wireless transmission to achieve a closed‐loop system to realize on‐demand treatment to improve healing outcome.^7^, ^18^

To realize the early detection of the wound status, many physicochemical markers, including temperature, pH, humidity, inflammatory factors, and toxins and enzymes secreted by bacteria, have been used as the indicators.^1^, ^7^ Among these, temperature, which is closely related to the inflammation and infection states at the wound site,^1^, ^19^, ^20^ is regarded as one of the most important and promising indicators.^21^ Abnormal wound‐temperature changes may be selected as an early predictor of infection before any other obvious symptom.^22^ To date, diverse tools for body temperature measurement, including infrared thermometers, colorimetric sensors, and electronic temperature sensors, have been developed.^1^, ^23^, ^24^ Among them, electronic temperature sensor shows many advantages, including high sensitivity, excellent accuracy, and easy operation in clinic. Recently, more attention is attracted in flexible electronic temperature sensor, which has obtained great achievements by the pioneered and outstanding studies.^24^, ^25^, ^26^ Such great progress has made it possible to design a smart wound dressing with real‐time wound‐temperature monitoring by an integrated flexible temperature sensor.

In addition, on‐demand therapy is another requisite for a smart wound dressing, which releases the therapeutic drugs according to wound pathological needs.^27^ Being aware of this, some researchers have attempted to develop advanced dressing systems to monitor wound status in real time and provide on‐demand treatments by combining a bioelectronics system with a responsive drug delivery system. Under this design principle, several wound dressings were reported that can guide the point‐of‐care treatment and provide feedback in a closed‐loop manner.^28^, ^29^, ^30^ These devices exhibit capabilities in capturing wound physiological signals for diagnosis and delivering therapeutic molecules on demand. In a recent pioneering study, Mostafalu et al.^29^ used a flexible pH sensor to monitor and indicate wound infection in real time. Moreover, a drug‐releasing system comprised of a thermo‐responsive drug‐release patch and a flexible microheater was also integrated into the bandage. Compared with traditional colorimetric detection of infection, such a flexible pH sensor‐based system, which transfers the endogenous infection signals into electrical signals, is more sensitive, intuitive, and accurate. However, it must be considered that an infection‐induced temperature increase may also trigger antibiotic release by a thermo‐responsive drug carrier. Therefore, a drug‐release system that is controlled by a separate exogenous stimulus is required. Because of the characteristics of cleanness, high spatiotemporal resolution, and remote controllability, light is widely used as a trigger to regulate the release of therapeutic molecules.^31^ Recently, by integrating light‐emitting diode (LED) units into a flexible electronic, many light‐based therapies have been performed in a wearable manner.^32^, ^33^

Herein, we report a smart flexible electronics‐integrated wound dressing, capable of monitoring wound temperature through an integrated sensor in real time as the early predictor of pathological infection. The on‐demand treatment capacity was implemented by integrating an ultraviolet (UV)‐responsive antibacterial hydrogel and UV‐LEDs into the system, by which antibiotics were released controllably in situ. The wound temperature was continuously monitored by the integrated sensor and transmitted to portable terminal equipment (e.g., smartphone) via Bluetooth. When the wound temperature continuously remained higher than a preset threshold value (e.g., 40 °C), an infected wound would be diagnosed, and the integrated UV‐LEDs were turned on to trigger antibiotic release in situ. As a demonstration of a flexible electronics‐integrated wound dressing, this proof‐of‐concept study provides a new strategy for overcoming the “Black Box” status of the wound‐healing process and implementing dynamic intervention treatment.

## 2 Results and Discussion

**Figure 1:**
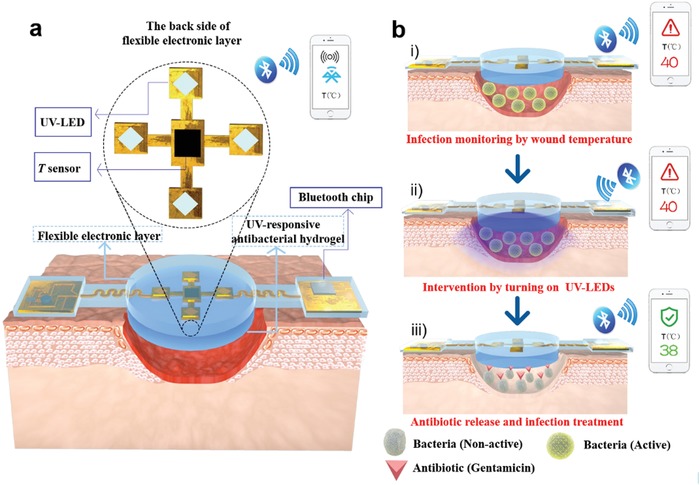
Schematics of the structures and working principles of the smart flexible electronics‐integrated wound dressing. a) The integrated system consists of a polydimethylsiloxane‐encapsulated flexible electronic layer and an UV‐responsive antibacterial hydrogel. The flexible electronic device is integrated with a sensor for monitoring temperature and four UV‐LEDs for emitting UV light (365 nm) to trigger the release of antibiotic from the UV‐responsive antibacterial hydrogel when needed; a Bluetooth chip is also integrated for wireless data transmission in real time. b) Conceptual view of the integrated system for infected‐wound monitoring and on‐demand treatment: i) real‐time monitoring of wound temperature and providing an alert of hyperthermia caused by infection; ii) turning on UV‐LEDs to trigger the release of antibiotics; iii) infection inhibition by the released antibiotics, resulting in decreased wound temperature.

The flexible electronics‐integrated wound dressing was engineered as a double‐layer structure with a woundplast shape to achieve conformal contact with the wound site (**Figure** **1**): 1) The upper layer was a flexible electronic layer, in which the power‐management module, circuit‐control module, and Bluetooth chip were distributed symmetrically on both sides, while a temperature sensor and four UV‐LEDs were embedded in the mid‐region. The connection circuit was designed as an s‐curved structure to ensure flexibility. In addition, the entire electronic system was encapsulated with flexible polydimethylsiloxane (PDMS) to ensure the system with good transparency, permeability, and biocompatibility; 2) the lower layer was an UV‐responsive antibacterial hydrogel of 3 mm thickness, in which gentamicin (GS) (a kind of aminoglycoside antibiotic with a killing effect toward *Staphylococcus aureus* (*S. aureus*)) was covalently grafted into a polyethylene glycol (PEG)‐based hydrogel through an UV‐cleavable linker and released upon UV irradiation at 365 nm; 3) the entire system was powered by an external battery. Temperature data read by the integrated sensor were wirelessly transmitted to a smartphone by Bluetooth, and the turn‐on of the UV‐LED was controlled by an app program. An inflammatory response caused by bacterial infection will induce wound overheat, normally 1–2 °C higher than that of normal skin. The fluctuation of wound temperature could be continuously monitored by a flexible temperature sensor because of its high sensitivity and seamless skin contact. Therefore, real‐time wound‐temperature monitoring should be a simple and effective diagnostic method for infection. Additionally, a smartphone installed with a controlled program was equipped with an integrated system to receive and process the collected data. Once the temperature was above the safe limit (40 °C in this study) for a given period, which was preset in the program to define an infection, the alarm and the intervention program would be activated. A subsequent decrease of the wound temperature could be used as a feedback to evaluate the treatment effect.

**Figure 2:**
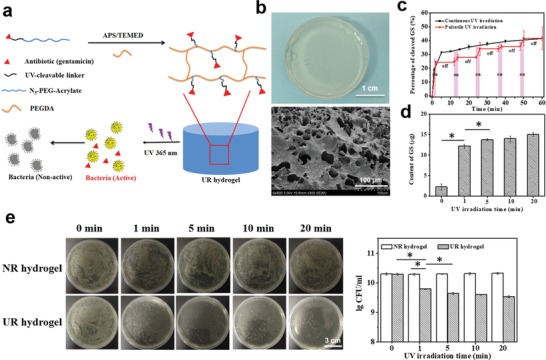
a) Scheme of the UV‐responsive antibacterial hydrogel, in which antibiotics (gentamicin, GS) are covalently grafted into the PEG‐based hydrogel through a UV‐cleavable linker and can be released under UV irradiation (365 nm, 110 w cm^−2^). b) Macroappearance and microstructure of the UV‐responsive hydrogels (UR hydrogel). c) GS cleavage profiles of the UV‐responsive polyprodrug (GS‐linker‐PEG‐Acrylate) under continuous UV irradiation (black line) and pulsatile UV irradiation (red line, 2 min on/10 min off for 5 circles). d) The amounts of the released GS from UR hydrogel under UV irradiation for different time; each group was tested in triplicate. e) In vitro antibacterial behavior of UR hydrogel, which was preirradiated with UV light for different time before being cocultured with *Staphylococcus aureus*. Nonresponsive hydrogels (NR hydrogel) were used as controls. Images (left) and the statistical analysis (right) of bacteria colony‐forming units (CFU) by a standard plate‐counting method.

In traditional antibacterial dressings, antibiotics are mainly physically loaded and released passively, which may result in antibiotic overuse and bacterial resistance. Therefore, to overcome these problems and realize on‐demand treatment, an UV‐responsive antibacterial hydrogel (UR hydrogel) was prepared as described in the scheme in **Figure** **2**a. We conjugated GS with azide‐PEG5000‐acrylate (N_3_‐PEG5000‐acrylate) by 1‐(5‐methoxy‐2‐nitro‐4‐prop‐2‐ynyloxyphenyl) ethyl N‐succinimidyl carbonate (a UV‐cleavable nitrobenzyl linker) to obtain the polyprodrug (GS‐linker‐PEG‐Acrylate), which was subsequently crosslinked by acrylate‐PEG acrylate (PEGDA) to form a hydrogel in the presence of ammonium persulfate (APS) and tetramethylethylenediamine (TEMED). The PEG‐based hydrogel without the UV‐cleavable linker, which has no UV‐responsive antibacterial property, was selected as the control (NR hydrogel). The detailed synthesis processes and the corresponding structure characterizations of the UV‐cleavable linker and the polyprodrug are, respectively, presented in Figures S1 and S2 (Supporting Information). In this system, UV light was selected as a stimulus because of its advantages of noninvasiveness, high efficiency, and high spatiotemporal resolution.^34^ Because the dimethoxynitrophenylethyl ester of the GS‐linker‐PEG‐acrylate is cleavable upon UV irradiation at 365 nm, this ensures that the GS can only be released from the hydrogel after UV irradiation. The UV‐responsive properties of the polyprodrug (GS‐linker‐PEG‐Acrylate) were proved by ^1^H NMR and UV–vis spectra (Figure S3a,b, Supporting Information). The results showed characteristic photolytic cleavage behavior of the 2‐nitrobenzyl ester, which was consistent with previous reports.^34^, ^35^ The percentage of the cleaved GS from the polyprodrug increased with the prolongation of the UV‐exposure time (black line in Figure 2c). Moreover, the GS cleavage exhibited an “on‐off” manner when pulsatile UV light was applied on the polyprodrug (red line in Figure 2c), proving that UR hydrogel composed of the polyprodrug should present an UV‐responsive release character for on‐demand GS release. The macroscopic photograph and microstructure of the hydrogel are presented in Figure 2b. The transparent appearance and porous microstructure ensured the hydrogel with good visibility and water absorption ability (Figure S4a, Supporting Information). The UV‐responsive release behavior of GS from the hydrogel was investigated (Figure 2d). Compared with the hydrogel without UV preirradiation (0 min), the amount of the released GS increased markedly with an increased UV‐irradiation time of 1 and 5 min. However, when the irradiation time was further increased to 10 and 20 min, there was no significant augmentation of the amount of the released GS. Because *S. aureus* is a common form of bacteria found in infected wounds, it was selected as model bacteria to evaluate the in vitro UV‐responsive antibacterial effect of the hydrogel by a standard plate‐counting method. The images and corresponding statistical results showed that for NR hydrogel group, no obvious difference in the number of bacteria colonies was detected however long the hydrogels were preirradiated (Figure 2e). The UR hydrogel group without UV irradiation showed a similar value to that of the NR groups. Whereas on UV‐light exposure of the UR hydrogels, a clear decrease in the number of bacteria colonies was observed, indicating a sustained release of GS from the UR hydrogel. When the exposure time of the UR hydrogels was increased to 10 min, no further decrease in the number of colonies was observed. These results were consistent with the GS‐release profiles (Figure 2d). The release of GS from UR hydrogel under UV irradiation and the UV‐responsive antibacterial effect proved that GS could be released from UR hydrogel in a controlled manner under the trigger of UV on the basis of wound need.

**Figure 3:**
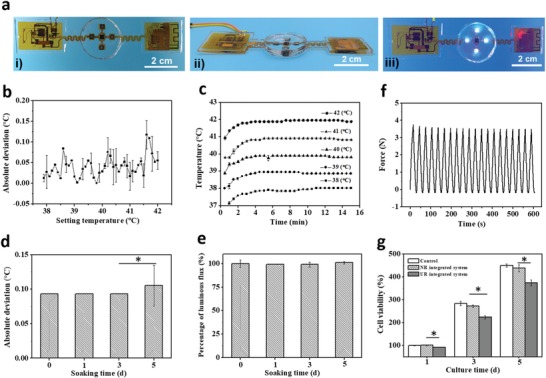
Characterizations of the flexible electronics‐integrated wound dressing. a) The optical images of i) the top view of the flexible electronic device, ii) the side view of the integrated system, and iii) the top view of the integrated system in working mode. b) Accuracy of the temperature sensor, which was defined as the absolute deviation between the recorded temperatures by the sensor and the corresponding setting temperatures in the range of 38–42 °C. c) Stability of the temperature sensor, which is presented by the temperature curves monitored continuously for 15 min at different temperature settings. d,e) Durability studies on the accuracy of the temperature sensor and luminous flux of the UV‐LEDs of the integrated system by soaking the integrated system into DMEM solution for different time intervals to simulate the humid wound environment. f) Fatigue test of the integrated system by stretching with a fixed strain of 6% for 20 cycles at a speed of 20 mm min^−1^. g) Biocompatibility of the integrated system by evaluating the viability of NIH 3T3 cells cultured in the extraction solution of the integrated system. DMEM and the extraction solution of the nonresponsive (NR) integrated system were used as controls.

The images of the flexible electronics‐integrated wound dressing are presented in **Figure** **3**a, the top view of which in working mode showed that the light of UV‐LEDs could cover almost the entire hydrogel. The integrated system was powered by an external battery (30 mm × 45 mm, 3.7 V and 2000 mAh) (Figure S5, Supporting Information). Regarding the monitoring function, the accuracy and durability of the temperature sensor are two critical parameters that may affect the assessment of infection. By inserting the PDMS‐encapsulated flexible electronic device into a thermostat with a temperature range from 38 to 42 °C (the temperature range of an infected wound), the mean absolute deviations between the recorded temperatures and the setting temperatures were profiled (Figure 3b). The temperature sensor exhibited excellent accuracy, with an absolute deviation of <0.15 °C within the entire test region. Additionally, as shown by the temperature–time curve (Figure 3c), the temperature sensor displayed a response time of <5 min and long‐time stability. Considering that the working environment of the system is complicated, the durability of the temperature sensor and UV‐LEDs was evaluated by immersing the device into Dulbecco's modified Eagle medium (DMEM) for different time intervals. The mean temperature deviation after soaking for 5 d remained <0.15 °C, and no obvious change was observed compared with the value of the original sensor (Figure 3d), indicating that the temperature sensor possesses good durability to meet the need for long‐term monitoring on the wound site. In addition, no significant decrease of the luminous flux was observed after immersing the device in DMEM solution for different time intervals (Figure 3e), proving that the property of the UV‐LEDs was quite stable even in a humid environment. Optical images of the integrated systems being soaked in DMEM are presented in Figure S6a (Supporting Information).

The mechanical property of the integrated system was evaluated by a tensile test. The device showed a Young's modulus of 3.5 MPa and good fatigue resistance when stretched at a maximum strain of 6% for 20 cycles (Figure 3f). The temperature sensor and UV‐LEDs continued to work during the cyclic tensile test (Figure S6b, Supporting Information), which demonstrates that this system has the ability to maintain its functions during routine deformation and movement.

When applying such a system in the wound site, it should neither be cytotoxic nor negatively affect cell growth. Therefore, we evaluated the cytotoxicity of the integrated systems. NIH 3T3 cells were selected as model cells and cocultured with the extraction solution of the integrated systems for 1, 3, and 5 d, with cells cultured with DMEM used as a control. Cell viability was assessed at each time interval using the 3‐(4, 5‐dimethylthiazol‐2‐yl)‐2, 5‐diphenyltetrazolium bromide (MTT) assay. The viabilities of the NIH 3T3 cells increased in all groups with an increase of culture time (Figure 3g), indicating that the integrated system did not affect NIH 3T3 cell proliferation. In addition, the viabilities of the cells irradiated by UV‐LEDs in situ were also evaluated (Figure S7, Supporting Information). No obvious difference was observed among the viabilities of the cells with or without UV irradiation, even for the group with 5 min irradiation. All the groups showed good cell proliferation behavior, proving that a short‐term UV irradiation has no obvious deterioration on cell viability and should be safety.

**Figure 4:**
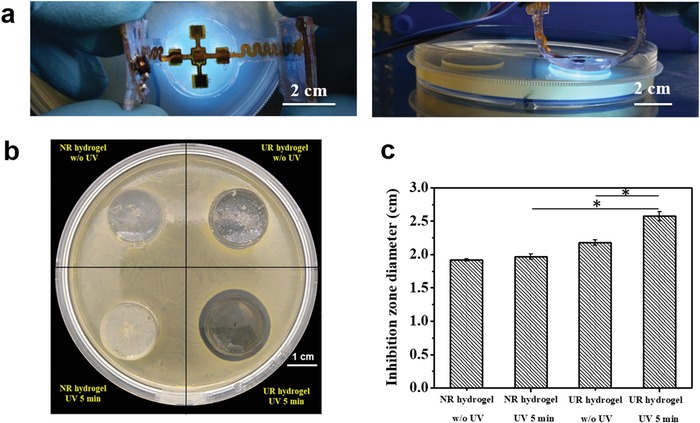
In vitro antibacterial property of the integrated system. a) Top view (upper) and side view (lower) of the integrated system for the in situ antibacterial test, the UV‐LEDs of which are turned on to trigger the release of antibiotics. b) Inhibition zones of the different integrated systems after in situ irradiation. c) Statistical analysis of inhibition zone diameters for different groups.

In the integrated system, UV irradiation was performed in situ by the embedded UV‐LEDs. To verify the antibacterial effect of the integrated system in situ, bacterial inhibition zone tests were performed. With the benefit of flexibility, the integrated system could be folded during the experiment (**Figure** **4**a). Under the control of the app program, the UV‐LEDs worked in situ to trigger the release of antibiotic from the hydrogel. There was a clear inhibition zone around the UR hydrogel after 5 min UV irradiation by the UV‐LEDs in situ (UR hydrogel UV 5 min). By contrast, for the sample without UV irradiation (UR hydrogel w/o UV) and NR hydrogels with or without UV irradiation (NR hydrogel UV 5 min or w/o UV), no clear inhibition zones were observed (Figure 4b). By the statistical analysis of the parallel experimental images (Figure S8, Supporting Information), the mean diameters of the inhibition zones were obtained (Figure 4c). It showed that in the group of UR hydrogel with 5 min UV irradiation, the mean diameter was significantly bigger than those of the other three groups, proving that the antibacterial property of the integrated system should be attributed to the released GS by UV irradiation. Moreover, no significant difference was observed between the NR hydrogels with or w/o UV irradiation, indicating that UV irradiation alone has no obvious antibacterial effect. Moreover, to exclude the influence of UV irradiation itself on bacteria viability, the bacteria solutions were irradiated by UV irradiation for different time, on which a hydrogel was placed to mimic the in vivo situation. The numbers of bacteria colonies after irradiating UV light for different time were evaluated by a standard plate‐counting method (Figure S9, Supporting Information). There was no significant decrease of bacteria colonies even for the sample being irradiated for 20 min, proving that UV irradiation itself had no obvious effect on killing bacteria in this study.

**Figure 5:**
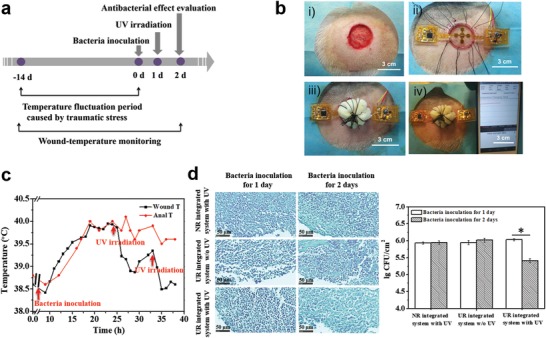
In vivo studies on infection monitoring and on‐demand treatment. a) Timeline of the animal experiments. b) Operational procedure: i) wound creation, ii) implantation of the integrated system, iii) pressure dressing, and iv) real‐time wound‐temperature monitoring. c) The curve of the recorded wound temperatures by the integrated system in real time. Anal temperature was measured as a reference. d) Gram stainings (left) and statistical analysis of bacteria densities (right) of the infected wounds treated by different groups.

To further verify the properties of real‐time monitoring and on‐demand treatment of the integrated wound dressing, we created a pig infected‐wound model, because the skin of this animal has considerable similarity with that of the human. According to the timeline of the animal experiment, we first created full‐thickness wounds on the pigs' back and implanted the UR integrated system with pressure dressing (**Figure** **5**a). Because incisions also give rise to a wound‐temperature increase, all the animals were fed until the wounds displayed an equilibrium temperature. Subsequently, an infected wound was created by inoculating the wound with a suspension of *S. aureus* at an initial density of 10^8^ CFU mL^−1^. The wound temperature was subsequently monitored in real time, and the anal temperature was measured as a reference. Once a continuous overheat was captured by the sensor, the preset program would emit an alarm of infection. Consequently, the LEDs were turned on to irradiate UV light. The operations of the animal experiments are presented in Figure 5b, including wound construction, implantation of the integrated system, pressure dressing, and in situ monitoring. Following wound creation, both the wound and anal temperatures clearly increased and reached the highest level (≈40 °C) within 7 d, and then returned to a normal level (≈38 °C) after ≈2 weeks (Figure S10a, Supporting Information). The healing process of a skin defect includes a series of consequent biochemical processes, including inflammation, new tissue formation, and remodeling.^36^ Among these processes, the noninfection inflammation will also induce wound overheating. Therefore, to eliminate the influence of wound temperature increase caused by the inflammatory reaction, the infected wound was created following the end of the inflammatory period (≈14 d after wound creation). Moreover, the temperature change of the system during UV‐LED working was also investigated and displayed in Figure S11 (Supporting Information). It is found that the temperature of the system increased during UV irradiation. However, it should be noticed that the temperature decreased very quickly to the room temperature (≈28 °C) within 2 min. Therefore, to eliminate the influence of UV irradiation on temperature fluctuation, the app was set to record the wound temperature 2 min after UV irradiation. The wound temperature monitored by the integrated system is presented in Figure 5c and the corresponding app interface for temperature monitoring is presented in Figure S12 (Supporting Information). Following bacteria inoculation, a rapid wound temperature increase was observed and a maximum temperature of 40 °C was detected. When the maximum temperature lasted for more than 6 h, which was defined as the occurrence of an infection, the UV‐LED lights were turned on to trigger antibiotic release. Subsequently, the wound temperature decreased gradually to 38.9 °C during the following 6 h. A reincrease of the wound temperature occurred 6 h after treatment, which could be diminished by repeated UV irradiation. The parallel studies were repeated in different pigs, and the curves with similar tendencies were obtained (Figure S13, Supporting Information). However, for the control group (UR‐integrated system w/o UV), the overheat induced by bacteria inoculation lasted in the entire period (Figure S14, Supporting Information). It is of note that although the anal temperature also increased on bacteria inoculation, the wound temperature displayed a more rapid response to the infection‐induced temperature fluctuation, further proving that wound temperature can be an effective predictor of infection. As the inflammatory response after injury will induce an obvious increase of wound temperature (Figure S10a, Supporting Information), how to distinguish an early infection from an injure‐caused inflammation should be considered. The temperature curve of an early infection model is displayed in Figure S10b (Supporting Information). Although the highest temperature of both models was almost same, the wound temperature increased to ≈40 °C within 48 h in early infection group, which was far less than the time needed in the injure‐caused inflammation model (≈7 d). The different temperature increasing profiles may be used to judge an early infection, indicating the good applicability of the system.

To evaluate the antibacterial effect of the integrated system, the wounds treated by different groups after bacteria inoculation for 1 and 2 d were harvested. To exclude the influences of the natural immune reactions of pigs and UV irradiation itself, UR‐integrated system w/o UV and NR‐integrated system with UV were also investigated as the controls. As the wounds gross images shown in Figure S15 (Supporting Information), the pustules were formed after bacteria inoculation for 1 d for all the groups. However, an obvious elimination was only observed on the wound site treated by UR‐integrated system with UV. From the Gram stainings in Figure 5d, there were many bacteria colonies in all the groups at day 1 after bacteria inoculation, but an obvious decrease was observed only in the group of UR‐integrated system with UV. By analyzing the parallel Gram stainings (Figure S16, Supporting Information), the densities of bacteria colonies were statistically quantified (Figure 5d, right). For NR‐integrated system with UV, no significant difference of bacteria density was observed at day 1 and day 2 after bacteria inoculation, indicating that UV light alone cannot inhibit infection effectively. In the case of UR‐integrated system w/o UV, there is no significant antibacterial effect either, meaning that without the aid of the released antibiotic, the natural immune reactions of pig are not able to inhibit infection. However, being different from the results of the control groups, the bacteria density of the wound treated by UR‐integrated system significantly decreased after UV irradiation. Meanwhile, the corresponding images of hematoxylin and eosin (HE) staining are presented in Figure S17a (Supporting Information). The inflammatory cells with round nucleus, the number of which indicates the inflammation situation of wound in some extent, were counted by Image J software and the statistical analysis of the densities of inflammatory cells for different groups is shown in Figure S17b (Supporting Information). For NR‐integrated system with UV group and UR‐integrated system w/o UV group, no significant difference was found between the numbers of inflammatory cells at day 1 and day 2, indicating that the inflammatory reaction did not fade away without the aid of antibiotic. While, differing from the control groups, the inflammatory cell density of the wound treated by UR‐integrated system significantly decreased after UV irradiation, showing a similar tendency with the densities of bacteria. It should be noted that the antibacterial effect was limited at the current stage. Presumably, by increasing the grafting degree of GS and UV irradiation time, a higher GS concentration could be obtained on wound site under UV irradiation, and thereby more effective bacteria inhibition should be achieved.

All these results proved that the integrated system was capable of monitoring wound status in real time, detecting bacterial infection in time, and providing on‐demand treatment. We demonstrated here that by combining emerging flexible bioelectronics with smart biomaterials, an integrated system with good flexibility, monitoring sensitivity, and durability and remote controllability can be applied to monitor wound status and provide intervention when necessary. Although this system was applied in a normal infection model in this study, it may also be used in the treatments of infection with drug‐resistant biofilm and diabetic ulcer with dysfunction of nervous system. The early detection and on‐demand therapy of this smart system should reduce the risks of the diabetes‐caused amputations. Moreover, the strategy proposed by this study may be extended to monitor other pathological signals in real time to realize precise diagnosis and on‐demand therapy.

## 3 Conclusion

We developed a smart flexible electronics‐integrated wound dressing capable of real‐time monitoring of wound temperature to diagnose infection at an early stage and providing on‐demand treatment by the UV‐triggered release of antibiotics. The integrated temperature sensor continuously collected wound temperatures, which were transmitted to a smartphone in real time by Bluetooth communication, and UV‐LEDs were used to remotely control the release of antibiotic in situ. In vitro studies demonstrated that the integrated system possesses good flexibility, excellent compatibility, and high monitoring sensitivity as well as long‐term durability in a humid environment. By creating an infection model in Bama mini pig, we verified the ability of the integrated system to monitor wound status in real time, predict infection at an early stage, and treat the infection on the basis of need. In this proposed system, we provided a novel strategy for overcoming the unknown status of the healing process by combining advanced biomaterials with flexible sensor technology. Herein, wound infection served as a model of pathology during the wound‐healing process and temperature was selected as one of the monitoring signs. Considering the complexity and diversity of the wound‐repair process, more pathological processes need to be investigated, and advanced systems integrated with different sensing components and therapeutic drugs need to be further developed for the treatment of different diseases.

## 4 Experimental Section

*Preparation of UV‐Responsive Hydrogel*: A UV‐responsive polyprodrug (GS‐linker‐PEG‐acrylate) was first synthesized by simply grafting GS in into N_3_‐PEG5000‐acrylate through a UV‐cleavable linker. The detailed synthesis process and characterizations of the UV‐cleavable linker and the UV‐responsive polyprodrug are, respectively, described in the Supporting Information. The UR hydrogels were prepared based on the crosslinking of GS‐linker‐PEG‐acrylate. In brief, CH_3_‐PEG5000‐acrylate and GS‐linker‐PEG‐acrylate were mixed at 7:1 (w/w) with a total mass concentration of 200 mg mL^−1^, and PEG2000‐DA (PEGDA) with different weight ratios of monomer (25%, 50%, 75%) were added as a cross‐linker. Following APS addition as the initiator and TEMED as the accelerator, the mixed solutions were degassed under nitrogen for 10 min, transferred to a polytetrafluoroethylene (PTFE) mold, and maintained at 70 °C for 30 min to obtain UR hydrogels. The hydrogels were washed with phosphate‐buffered saline (PBS) to remove unreacted residues and monomers. In addition, the hydrogels without GS‐linker‐PEG‐acrylate were prepared as a control following the same procedure (termed NR hydrogel).

*Scanning Electron Microscopy (SEM) Characterization of the UV‐Responsive Hydrogel*: UR hydrogels were first dehydrated in graded ethanol (10%, 25%, 50%, 75%, 100%), then replaced with tertiary butanol. Following freezing in liquid nitrogen for 10 min and lyophilization, the microstructures of the hydrogels were observed by SEM (HITACHI S‐4800, Hitachi, Japan).

*Gentamicin Release from the Hydrogel upon UV Irradiation*: UR hydrogels prepared in 96‐well plates (diameter 8 mm, thickness 6 mm) were preirradiated using a UV spot‐light source (365 nm, 110 mW cm^−2^, UVEC‐4II, LAMPLIC) for 0 1, 5, 10, or 20 min, then immersed into PBS (1 mL, pH 7.4) at 37 °C with continuous shaking. After 3 d, the supernatants were collected and the amount of GS released from the hydrogels were quantified by ultraviolet spectrum (UV‐2550, Shimadzu, Japan). Hereby, the collected GS solution was mixed with the derivatization reagent and 2‐propanol in the same volume ratio at room temperature for 30 min and the UV–vis absorbance was determined at 332 nm.^37^

*In Vitro UV‐Responsive Antibacterial Assay*: Following preirradiation using a UV spot‐light source (365 nm, 110 mW cm^−2^, UVEC‐4II, LAMPLIC) for 0, 1, 5, 10, or 20 min, UR hydrogels and NR hydrogels (diameter 8 mm, thickness 6 mm) were transferred to 48‐well plates, into which *S. aureus* (ATCC 25923) solutions with a concentration of 10^6^ CFU mL^−1^ were added. The blank *S. aureus* solution was used as a control. Following 24 h incubation at 37 °C, the bacterial suspensions were collected and diluted with PBS by different factors (10^1^ to 10^8^), and diluent (100 µL) was coated on an agar plate and incubated at 37 °C overnight to obtain a single colony. The bacteria colony‐forming units per milliliter were counted and plotted in a log scale.

*Fabrication of the Flexible Electronic Layer*: The flexible printed circuit board was processed using polyimide as the substrate. The circuit board was designed with a woundplast shape, in which a power‐management module and circuit‐control module were distributed symmetrically on both sides and the temperature sensor and UV‐LEDs were integrated in the center of the circuit board. Before usage, the flexible printed circuit board was encapsulated with PDMS. In brief, first a thin PDMS layer was coated on the PTFE template and presolidified for 5 min at 60 °C, then the reverse side of the flexible printed circuit was bonded with presolidified PDMS, and finally sufficient PDMS was added for encapsulation. The PDMS‐encapsulated device was incubated at 60 °C in an air oven for 20 min to ensure complete solidification of the PDMS.

*Integration of the Flexible Electronic and UV‐Responsive Hydrogel*: Hydrogel of the same size as the circular region of the flexible electronic layer was prepared and amalgamated with the flexible electronic layer during the PDMS solidification procedure to obtain the integrated system. Briefly, the flexible electronic was casted with PDMS precursors at 60 °C for 5 min. Then, the hydrogel was subsequently placed on the semisolidified PDMS layer, which shows strong viscidity to adhere. After further 10 min solidification of PDMS, the two layers were combined to form the integrated system.

*Accuracy and Stability Tests of the Temperature Sensor*: The accuracy of the temperature sensor of the flexible electronic device was evaluated using a Benchtop Temperature Test Chamber (accuracy of 0.1 °C, SH‐261, ESPEC, Germany). Briefly, the chamber temperature was increased at a rate of 0.1 °C min^−1^ from 38 to 42 °C, and the temperatures measured by the integrated system were recorded accordingly. The deviation between the recorded temperature and the setting temperature was defined as the temperature sensor accuracy at each temperature point. In addition, the stability, another important parameter of a temperature sensor for long‐time monitoring, was tested by placing the integrated device in the chamber with temperature settings of 38, 39, 40, 41, and 42 °C for a 15 min continuous monitoring.

*Durability Evaluation of the Temperature Sensor and UV‐LEDs*: To simulate the humid wound environment, the integrated systems were immersed into DMEM, and the accuracy of the temperature sensor and the luminous flux of the UV‐LEDs were evaluated at 0, 1, 3, and 5 d by a Benchtop Temperature Test Chamber and a luminous flux tester (LS‐2, Hangzhou Scirise Optoelectronic Co. Ltd., China), respectively.

*Mechanical Test of the Integrated System*: The tensile properties of the integrated system were determined using a universal mechanics test machine (CMT 4204, Shenzhen SANS Test Machine Co. Ltd., China). The dumbbell‐shaped sample with a gauge length of 75 mm and a width of 5 mm was tested at room temperature with a stretch rate of 20 mm min^−1^. The Young's modulus was calculated from the slope of the stress–strain curve within a strain of 5%. Cyclic tensile tests were also performed by sequentially loading and unloading at a rate of 20 mm min^−1^ for 20 cycles with a maximum strain of 6%.

*Cytotoxicity Assay*: The cytotoxicity of the integrated device was evaluated by determining the viability of NIH 3T3 cells cultured with the extraction medium. Briefly, the UR‐integrated system and NR‐integrated system were soaked into DMEM at 37 °C for 24 h with a concentration of 0.2 g mL^−1^ (weight of device/volume of DMEM) to collect the extraction solution. The cells were seeded in a 96‐cell plate at a density of 7000 cells well^−1^ and cultured with the extraction solution for 1, 3, or 5 d. Cells cultured in pure DMEM were used as a control. At each time interval, the culture medium was removed, to which MTT (200 µL, 0.5 mg mL^−1^) was added and incubated for 4 h at 37 °C. Subsequently, dimethyl sulfoxide (50 µL) was added to dissolve the dark blue formazan crystals generated by mitochondrial dehydrogenase in living cells. Cell viability was evaluated by determining the absorbance at 570 nm using a microplate reader (Model 200 PRO, TECAN, USA). Five repeats were conducted for each group.

*In Situ UV‐Responsive Antibacterial Assay of the Integrated System: S. aureus* suspensions (500 µL) with a concentration of 10^8^ CFU mL^−1^ were seeded evenly over solid agar culture plates, on which the UR‐integrated systems were subsequently placed. The UV‐LEDs were controllably lighted in situ for 0 and 5 min. The upper layer of the flexible electronic device was removed after UV irradiation and the plate was incubated at 37 °C for 24 h. Similarly, a NR‐integrated system was selected as a control. The antibacterial effects were analyzed by an inhibition zone test, three parallel samples were tested for each group, and the mean diameters of inhibition zones were statistically quantified using Image J software.

*Wound Infection Model Creation, Monitoring, and Treatment*: Animal experiments were performed according to the Guidelines for Animal Care and Use Committee of Zhejiang University. To establish animal wound‐infection models, Bama mini pigs (7–9 kg, female, obtained from Jiagan Biotechnology Co., Ltd., Shanghai, China) were acclimatized for one week before the experiments. Following anesthesia by xylazine hydrochloride injection (3.5 mg kg^−1^ of body weight), the hair was shaved and the surface was sterilized using iodophor. A full‐thickness wound of 3 cm diameter was created on the dorsal surface of each pig and a polypropylene ring of the same size was placed in the wound to prevent wound shrinkage. Following pressure dressing, the pigs were fed for another two weeks. Subsequently, the wounds were inoculated with a suspension of *S. aureus* (2 mL) with an initial density of 10^8^ CFU mL^−1^ and then were covered with an UR‐integrated system and wrapped by gauze again. The wound temperature was subsequently monitored continuously and transmitted to a smartphone by Bluetooth. The anal temperature was measured as a control using an electronic thermometer. When an overheated wound temperature was detected, the UV‐LEDs were turned on to emit UV irradiation discontinuously. The wound with the bacteria inoculation but without UV irradiation was investigated as a control (UR‐integrated system w/o UV). NR‐integrated system with UV was also studied as a control to exclude the possibility that bacteria were killed by UV light. Two infected wounds for each group were created and harvested separately: One was harvested after bacteria inoculation for 1 d and the other was harvested after bacteria inoculation for 2 d. The bacteria densities were calculated from the images of Gram stainings by Image J software. The inflammation situation after bacteria inoculation for 1 and 2 d for different groups was also evaluated, respectively, by HE stainings, and the density of inflammatory cell was calculated from the images of HE stainings by Image J software. An early infection model was also tested by inoculating bacteria (2 mL, with an initial density of 10^8^ CFU mL^−1^) as soon as the wound was created, and the subsequent temperature was monitored continuously by the system.

*Statistical Analysis*: Data are expressed as mean ± standard deviation (SD). Statistical analysis was performed by two‐tailed Student's *t*‐tests between two groups or by one‐way ANOVA between more groups. The star (*) indicates the level of statistical significance (*p*) < 0.05.

## Conflict of Interest

The authors declare no conflict of interest.

## Supporting information

Supporting Information
