# Supplementary material for: Smart Flexible Electronics‐Integrated Wound Dressing for Real‐Time Monitoring and On‐Demand Treatment of Infected Wounds
Source: Adv Sci (Weinh). 2020 Jan 10;7(6):1902673. doi: 10.1002/advs.201902673 (PMC7080536; doi:10.1002/advs.201902673)
Supplement: Supplementary file 1 — Supporting Information [file ADVS-7-1902673-s001.pdf]

Copyright WILEY-VCH Verlag GmbH & Co. KGaA, 69469 Weinheim, Germany, 2020.

ADVANCED SCIENCE
Open Access

Supporting Information

for *Adv. Sci.,* DOI: 10.1002/advs.201902673

Smart Flexible Electronics-Integrated Wound Dressing for Real-Time Monitoring and On-Demand Treatment of Infected Wounds

Qian Pang, Dong Lou, Shijian Li, Guangming Wang, Bianbian Qiao, Shurong Dong,* Lie Ma,* Changyou Gao, and Zhaohui Wu*

Supporting information

Smart Flexible Electronic-integrated Wound Dressing for Real-time Monitoring and On-demand Treatment of Infected Wounds

Qian Pang[#], Dong Lou[#], Shijian Li, Guangming Wang, Bianbian Qiao, Shurong Dong*, Lie Ma*, Changyou Gao and Zhaohui Wu*

Q. Pang, D. Lou, B.B. Qiao, Prof. L. Ma, Prof. C.Y. Gao

MOE Key Laboratory of Macromolecular Synthesis and Functionalization, Department of Polymer Science and Engineering, Zhejiang University, Hangzhou, 310027, China.

E-mail: liema@zju.edu.cn;

G.M.Wang, Prof. S.R. Dong

Key Laboratory of Advanced Micro/Nano Electronic Devices & Smart Systems of Zhejiang, College of Information Science and Electronic Engineering, Zhejiang University, Hangzhou 310027, China.

E-mail: dongshurong@zju.edu.cn

Dr. S.J. Li, Prof. Z.H. Wu

College of Computer Science and Technology, Zhejiang University, Hangzhou 310027, China.

E-mail: shijianli@zju.edu.cn

# these authors contributed equally to this work

Table of Contents

Supplementary Methods **Page**

1. Synthesis and Characterizations of 1-(5-Methoxy-2-nitro-4-prop-2-ynyloxyphenyl) ethyl N-succinimidyl Carbonate (UV-cleavable linker) 3

2. Synthesis and Characterizations of UV-responsive Polyprodrug (GS-linker-PEG-Acrylate)...... 4

3. The UV-responsive Properties Assay of GS-linker-PEG-Acrylate 4

4. Characterizations of UV-responsive Antibacterial Hydrogel 6

5. In Vitro Viabilities of NIH 3T3 Cells after being Pre-irradiated by UV in situ 7

6. In Vivo Monitoring of Wound Temperature........ 7

Supplementary Figures and Legends S1-S17 8

.... 18

References........ 18

1. Synthesis and Characterizations of 1-(5-Methoxy-2-nitro-4-prop-2-ynyloxyphenyl) ethyl N-succinimidyl Carbonate (UV-cleavable linker)

The UV-cleavable linker was prepared according to the previously reported protocol with minor modification (Figure S1 a).[1] Firstly, 4-hydroxy-5-methoxy-2-nitroacetophenone (Molecule 1, 0.5 g, 2.36 mmol) and potassium carbonate (0.476 g, 3.64 mmol) were dissolved to a 25 ml dry acetonitrile solution, and then 3-bromoprop-1-yne (Moleculer 2, 0.28 ml, 3.64 mmol) was subsequently added. The solution was refluxed at 100 ºC for 3 h under nitrogen atmosphere and concentrated using a rotary evaporator. After that, the solid was redissolved with HCl solution (0.2 M) and extracted with chloroform. The organic layer was collected and dried over $MgSO_4$, and the solution was removed under a reduced pressure to obtain the solid product (Molecule 3). The structure of the product was characterized by ^{1}H NMR (500 MHz, $CDCl_3$, Avance III HD, Bruker, CH) (Figure S1 b) and FTIR (Nicolet 6700, Themo Fisher scientific LLC, USA) (Figure S1 e). Then, the product (0.57 g, 2 mmol) and sodium tetrahydroborate (0.69 g, 1373mmol) were continuously added into a mixed solution of THF and methanol (1:2 v/v) on an ice bath and stirred for another 3 h. After concentrating the solution on a rotary evaporator, the solid was redissolved with HCl solution (0.2 M) and extracted with chloroform. The organic layer was collected and dried over $MgSO_4$, and the solution was removed under a reduced pressure to obtain the solid product (Molecule 4). The structure of the product was characterized by ^{1}H NMR (500 MHz, $CDCl_3$) (Figure S1 c) and FTIR (Figure S1 e). Finally, the product (0.55 g, 2.19 mmol) obtained from last step and N, N'-Disuccinimidyl carbonate (1.68 g, 6.57mmol) was further dissolved to a 25 ml dry acetonitrile solution in the presence of triethylamine (1mL, 4 mmol). The solution was stirred at room temperature for 5 h under nitrogen atmosphere then concentrated using a rotary evaporator. After that, the solid was redissolved with HCl solution (0.2 M) and extracted with chloroform. The organic layer was washed with saturated $NaHCO_3$ and dried over $MgSO_4$. The final product was concentrated under reduced pressure and dried in vacuum, which was the UV-cleavable linker. The structure of the product was characterized by ^{1}H NMR (500 MHz, $CDCl_3$) (Figure S1 d) and FTIR

(Figure S1 e). The chemical shifts at 2.6 ppm and 2.8 ppm in NMR spectrum (Figure S1 d), which belong to the typical peaks of alkynyl group and succinimidyl carbonate group of the UV-cleavable linker, proved its successful synthesis.

2. Synthesis and Characterizations of UV-responsive Polyprodrug (GS-linker-PEG-Acrylate)

The synthesis of the UV-responsive polyprodrug was described in Figure S2 a. In brief, gentamicin (GS) was first reacted with the UV-cleavable linker at a molar ratio of 1.2:1 in the mixed solvent containing triethylamine (THF/H_2O=2:1 v/v). The mixed solution was stirred under nitrogen atmosphere for 12 h at room temperature. Then the reaction solution was directly added into azide-PEG-acrylate (N_3-PEG5000-Acrylate, Mw=5000) solution (2% w/w in THF) in the presence of copper (II) sulfate (0.1 M/L, 5% mol of alkynyl group) and L-ascorbic acid (0.1 M/L, 10% mol of alkynyl group). After stirring under nitrogen atmosphere for another 12 h at 60 °C, the reaction solution was cooled and dialyzed against distilled water for 3 days to remove the unreacted molecular (MWCO: 1000). Finally, the dialysis solution was lyophilized to obtain the UV-responsive polyprodrug (GS-linker-PEG-Acrylate), the structure of which was characterized via ^{1}H NMR spectra (500 MHz, D_2O) and FTIR. The chemical shifts at 6.75 ppm (c) and 8.0 ppm (d) in NMR spectrum (Figure S2 c), which belong to the typical peaks of benzylic proton of linker and triazole ring formed by giclk reaction, proving its successful synthesis. The corresponding FTIR analysis was showed in Figure S2 d.

3. The UV-responsive Properties Assay of GS-linker-PEG-Acrylate

To analyze the UV-responsive cleavage behavior, the structures of GS-linker-PEG-Acrylate before and after UV irradiation were studied through ^{1}H NMR spectra. In brief, GS-linker-PEG-Acrylate solution was first irradiated by a UV spot light source (365 nm, 110 mW/cm², UVEC-4II, LAMPLIC) for 2 h, and then dialyzed against distilled water for 3 days to remove the cleaved molecule. The product was lyophilized and characterized by ^{1}H NMR (500 MHz, D_2O). From the

result (Figure S3 a), the peak at δ 6.75 ppm, which belongs to benzylic proton of linker, disappeared after UV irradiation, indicating the successful cleavage of dimethoxynitrophenylethyl ester. The photo-cleavage reactions of GS-linker-PEG-Acrylate with different UV irradiation time were also traced by UV-vis spectra (Figure S3 b). The typical absorption peaks at 304 and 333 nm of dimethoxynitrophenylethyl ester showed a time-dependent decrease and an overall redshift, indicating that the polyprodrug undergoes a photochemical breakage of 2-nitrobenzyl ester. These results were consistent with previous reports on the characteristic of photolytic cleavage of 2-nitrobenzyl ester.[2] To monitor the cleavage kinetics of GS-linker-PEG-Acrylate, after being continuously irradiated by UV light for different time, the solutions were transferred to ultrafiltration centrifuge tubes (MWCO: 3000) and then centrifuged at 12,000 rpm for 35 min. The filtrate was collected and reacted with derivatization reagent. As there is no conjugated structure in GS, the GS solution had to be derived according to the method previously reported by Tang et al.[3] The principle was based on o-phthaldialdehyde reacting with the primary amino group of GS to form chromophoric products, whose absorbance was measured at 332 nm. Briefly, the derivatization reagent was formulated by adding 250 mg of o-phthaldialdehyde, 6.25 mL of methanol and 300 μL of 2-mercaptoethanol to 56 mL of sodium borate in distilled water. The mixed solution could be used after storing in a brown bottle in a dark chamber for at least 24 h, and the content of GS was subsequently measured by a microplate reader (Model 200 PRO, TECAN, USA) at 332 nm. The cumulative cleavage percentage of GS was obtained (black line in Figure 2c). To further confirm the UV-responsive release character of the polyprodrug, pulsatile UV irradiation was carried out for 5 circles (2 min light on/ 10 min light off), and the samples were collected at time points of 2, 12, 14, 24, 26, 36, 38, 48, 50, 60 min, respectively. The absorbance of GS at each time point was subsequently measured as described above. All experiments were carried out in triplicate.

4. Characterizations of UV-responsive Antibacterial Hydrogel

Swelling Ability Assay*:* Hydrogels with different contents of cross-linker (25 %, 50 %, 75 %) were dried at 50 ºC for 6 h and weighed to obtain the initial weight (W_0). Then the samples were soaked into PBS (pH 7.4) at 37 ºC. At each pre-determined time intervals, the samples were weighed again (W). The swelling degree was calculated using the following equation:

Swelling degree (%) = $\frac{W - W_0}{W_0}$ ×100%. Each group was tested in triplicate. The results were showed in Figure S4 a. All hydrogels can reach swelling equilibrium within 2 h, and the swelling ability decreased with the increasing cross-linker contents. However, the swelling degree can still reach to 500% even with high crosslinking degree, indicating a good ability to absorb wound fluid and exudates.

Mechanical Property Test: The three kinds of hydrogels (diameter 8 mm, thickness 6 mm) were compressed using a universal mechanics test machine (INSTRON 5543A, UK) at a compressing rate of 0.5 mm/min. The compression modulus of each sample was calculated as the slope of straight line section of the stress-strain curve (1%-3%). Three parallel samples were measured for each group. Figure S4 b described the results. Considering swelling ability and mechanical property of hydrogel, hydrogels with 50% mass ratio of cross-linker were selected as the lower layer of the smart integrated system.

Cytotoxicity Assay: The cell viability of UV-responsive hydrogel was studied using the same procedure with the integrated system described in experimental section. Accordingly, non-responsive hydrogel was used as a control. Each group was conducted with five repeats. The results were showed in Figure S4 c.

5. In Vitro Viabilities of NIH 3T3 Cells after being Pre-irradiated by UV in situ

To evaluate the effect of UV irradiation to celll viability, NIH 3T3 cells were pre-irridiated by UV-LED for 0 s, 30 s, 60 s and 5 min. The cell vibilities were analyzed at 1, 3 and 5 day for each group using MTT method. The results were showed in Figure S7.

6. In Vivo Monitoring of Wound Temperature

After a full-thickness wound on the back of pig was created, the UV-responsive integrated system was implanted into the wound with a pressure dressing. The wound temperature was subsequently monitored and transmitted to the smartphone by Bluethooth in the next 2 weeks. The app interfaces with infection and without infection were shown in Figure S12 and the normal wound temperature data with the increase of healing time were described in Figure S10 a.

Supplementary Figures and Legends S1-S17

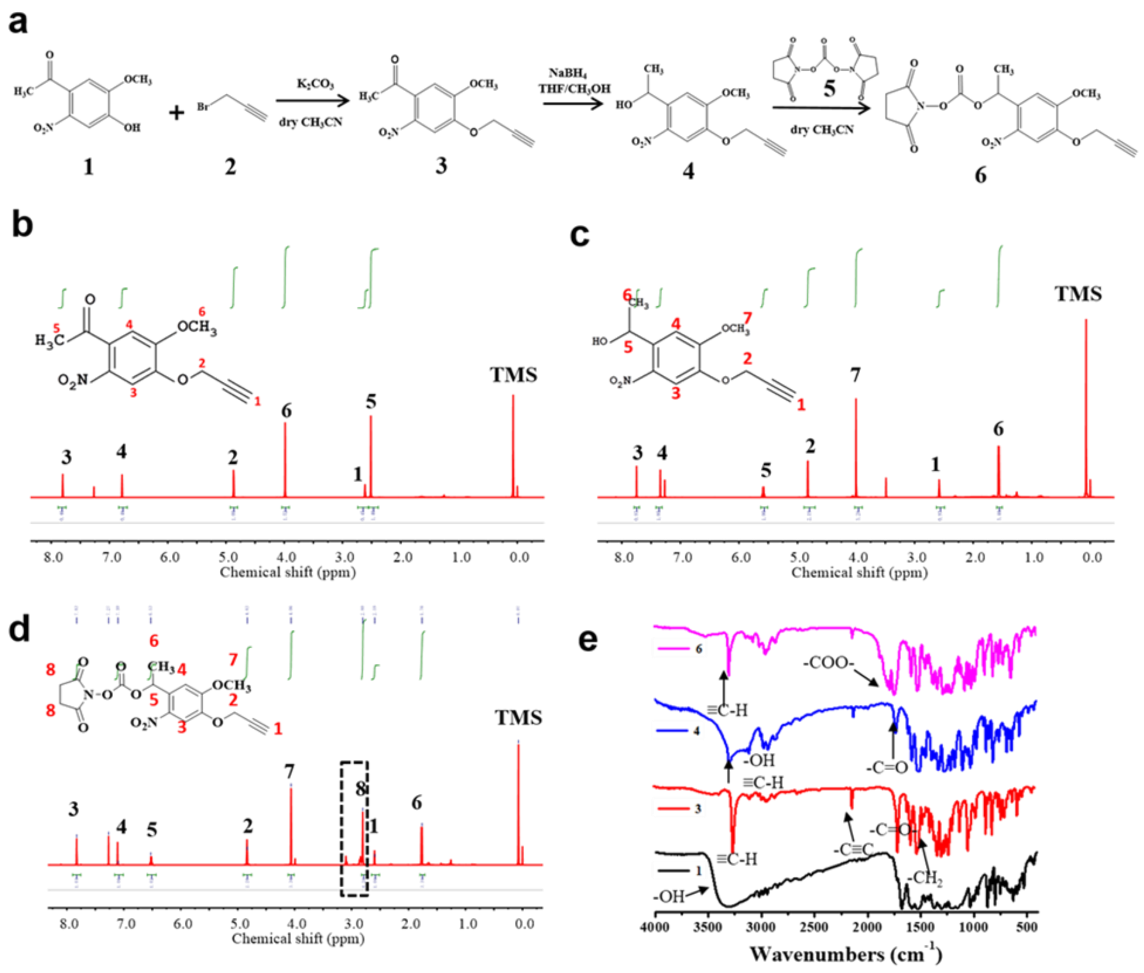

Figure S1. Synthesis route (a) and structure characterizations (b-e) of the UV-cleavable linker. ^{1}H NMR characterizations of molecule 3 (b), molecule 4 (c) and molecule 6 (d), and corresponding FTIR analysis of each product (e).

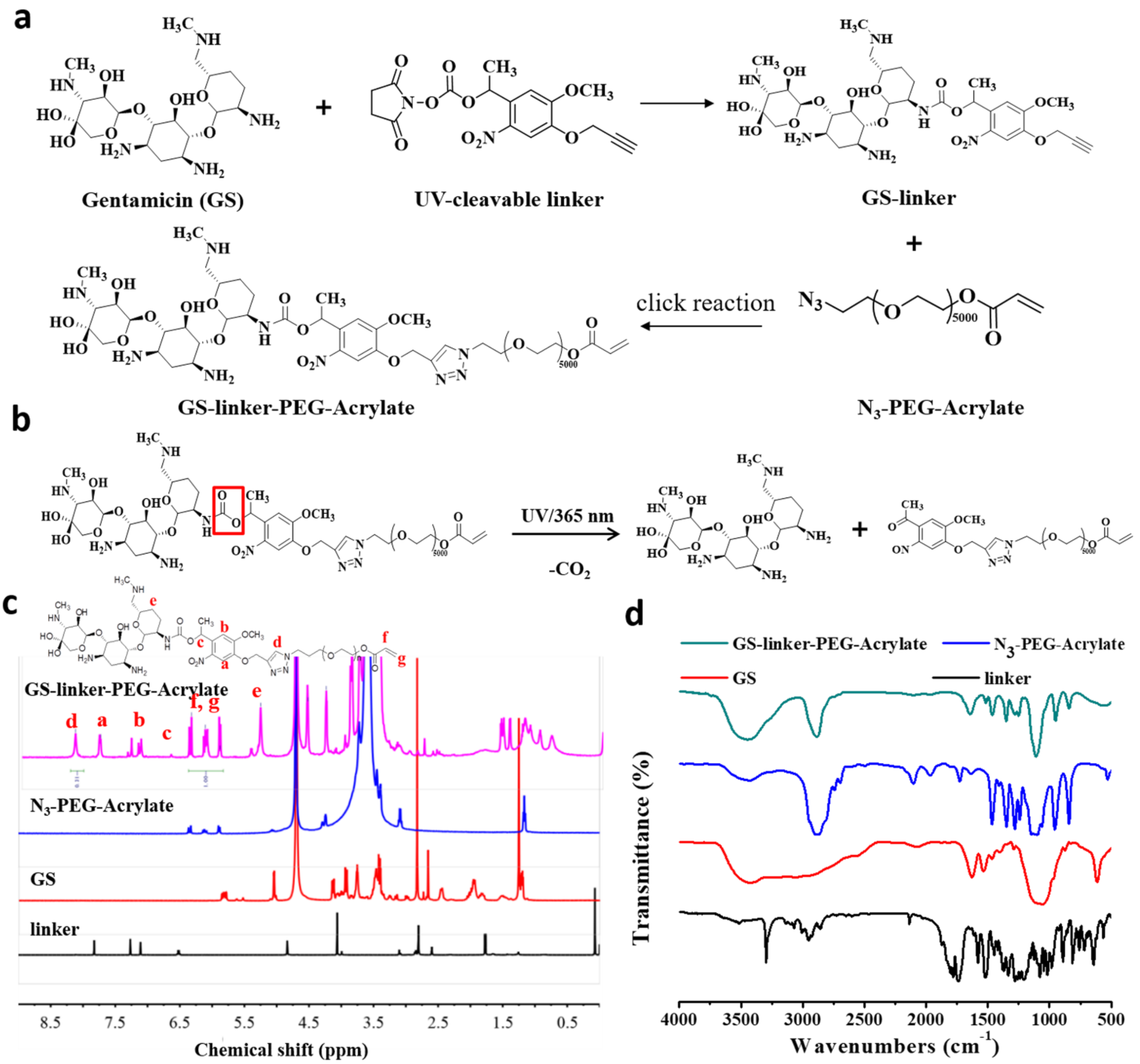

Figure S2. Synthesis route (a) and photo-cleavable mechanism (b) of the UV-responsive polyprodrug (GS-linker-PEG-Acrylate). ^{1}H NMR characterizations (c) and corresponding FTIR analysis (d) of GS-linker-PEG-Acrylate.

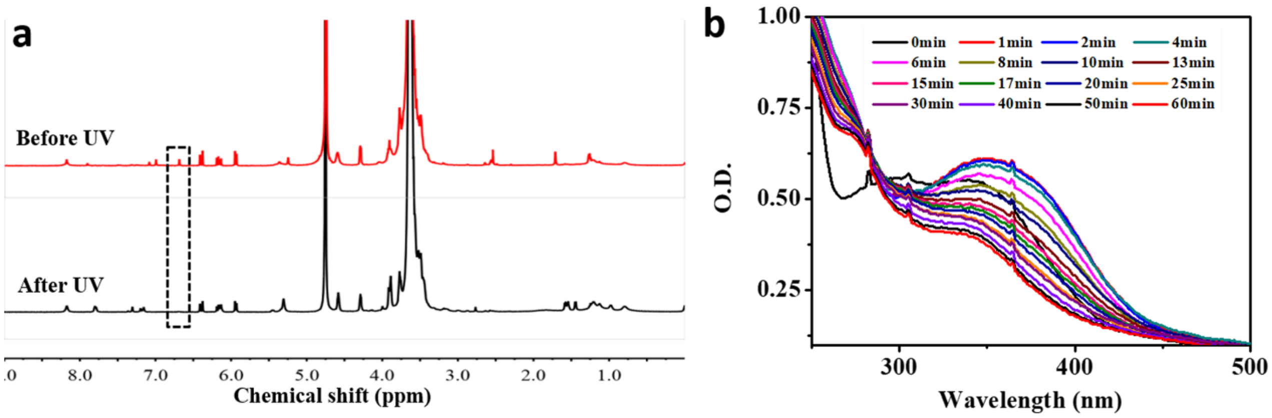

Figure S3. UV-cleavable properties evaluation of GS-linker-PEG-Acrylate. a) 1H NMR structure analysis of GS-linker-PEG-Acrylate before and after UV irradiation; b) UV-vis spectra of GS-linker-PEG-Acrylate with the increasing UV irradiation time;

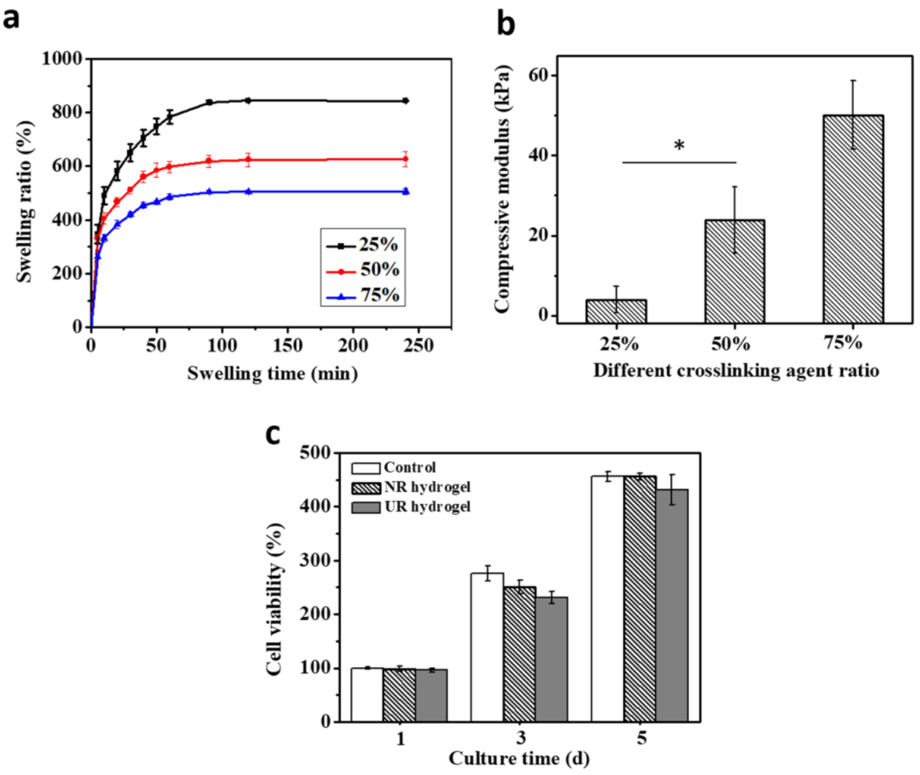

Figure S4. Characterizations of the UV-responsive antibacterial hydrogel. a) Swelling ability of the hydrogels with different content of cross-linker. b) Mechanical properties of different hydrogels. c) Biocompatibility evaluation of the UV responsive hydrogel by investigating the viabilities of NIH 3T3 cells cultured by the extracting solution of the hydrogel. DMEM and the extracting solution of NR hydrogel were used as controls.

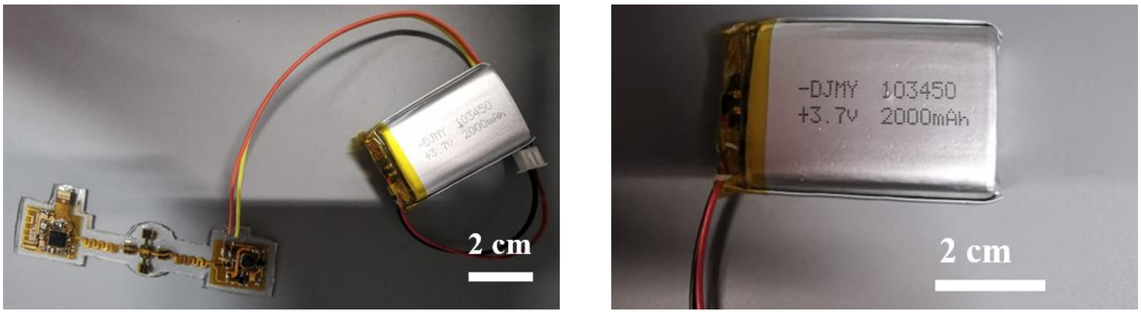

Figure S5. Optical photograpies of the integrated system powered by an external battery (left) and the battery with higher mangification (right, 30 mm × 45 mm, 3.7 V and 2000 mAh).

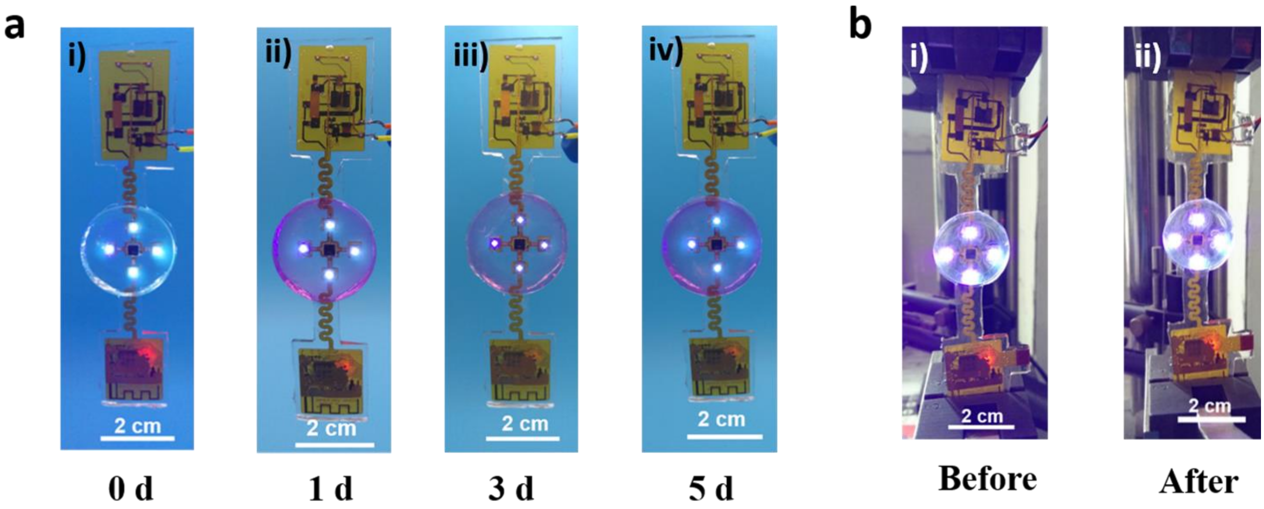

Figure S6. Optical images of the integrated system in a working mode a) after being soaked in DMEM for different time and b) before and after cyclic tensile test.

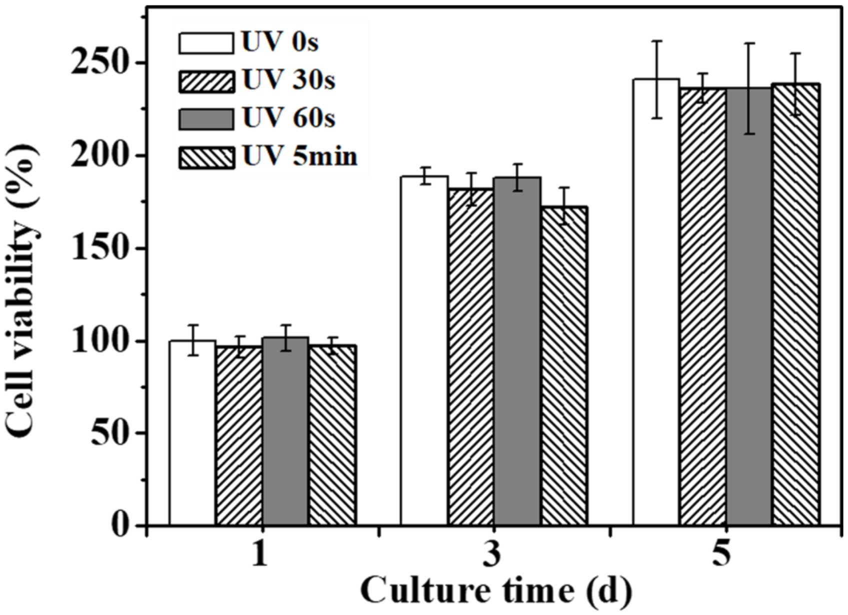

Figure S7. Cell viabilities of NIH 3T3 cells after pre-irradiating by UV-LED in situ, then cultured for different time.

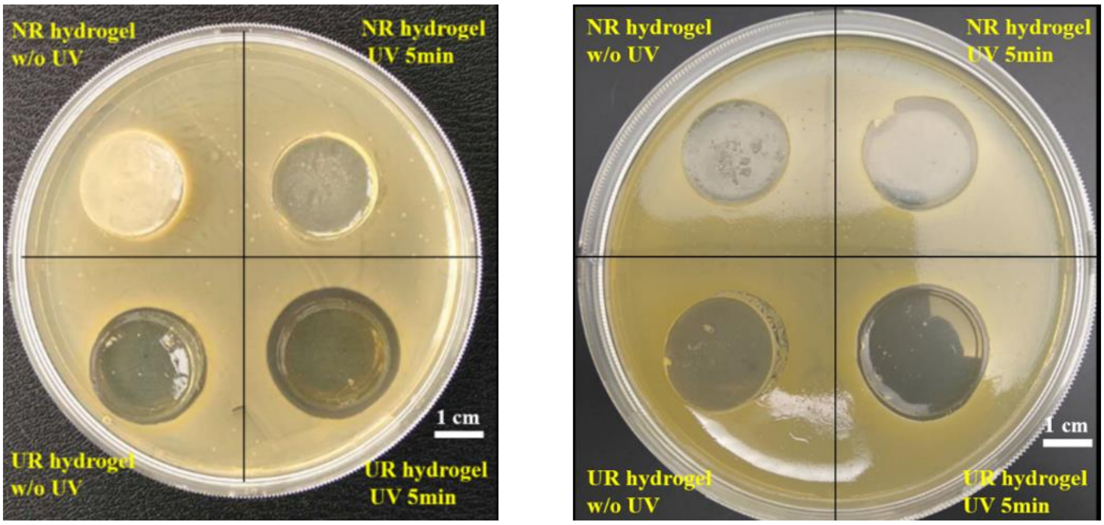

Figure S8. The parallel inhibition zone images of the different integrated systems after in situ irradiation.

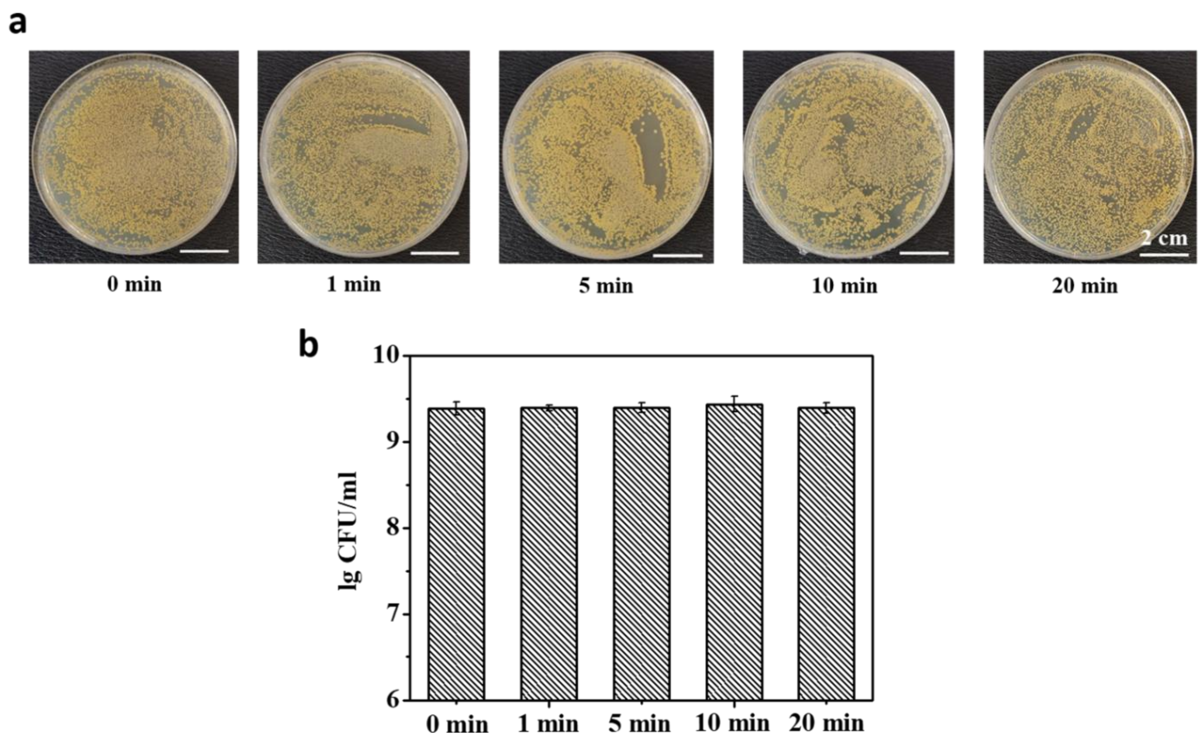

Figure S9. In vitro evaluation of bacteria viability after irridiating the bacteria solution with UV (365 nm, 110 w cm^{-2}) in situ for different time by a standard plate-counting method. Images (a) and the statistical analysis (b) of bacteria colony-forming units (CFU) by a standard plate-counting method.

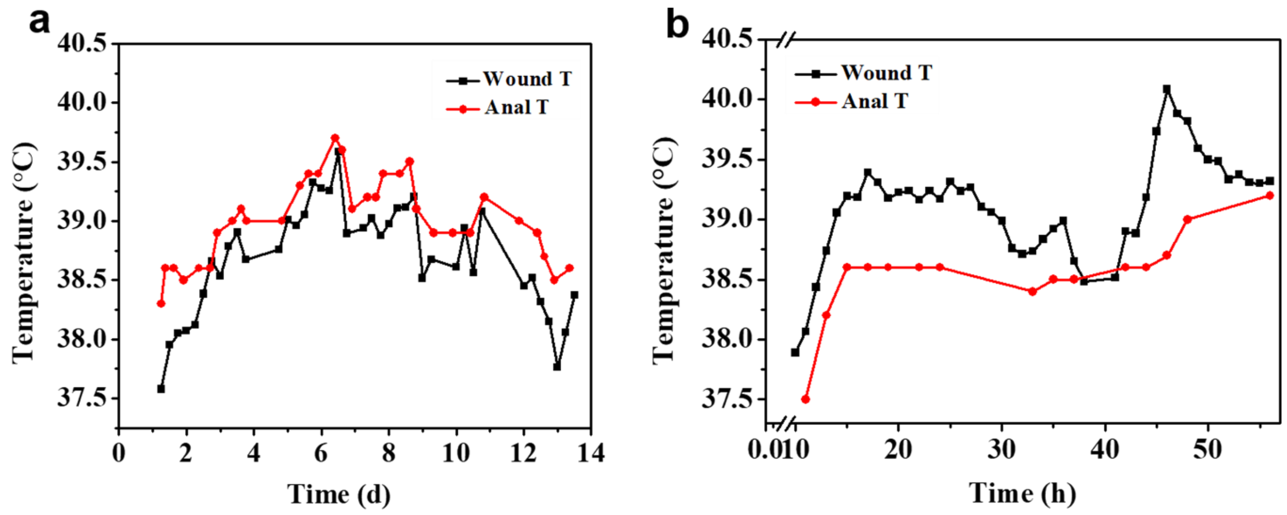

Figure S10. Temperature curves of normal wound with the increase of healing time (a) and ealy infection wound by inoculating bacteria as soon as wound was created (b). Anal temperature was measured as a reference.

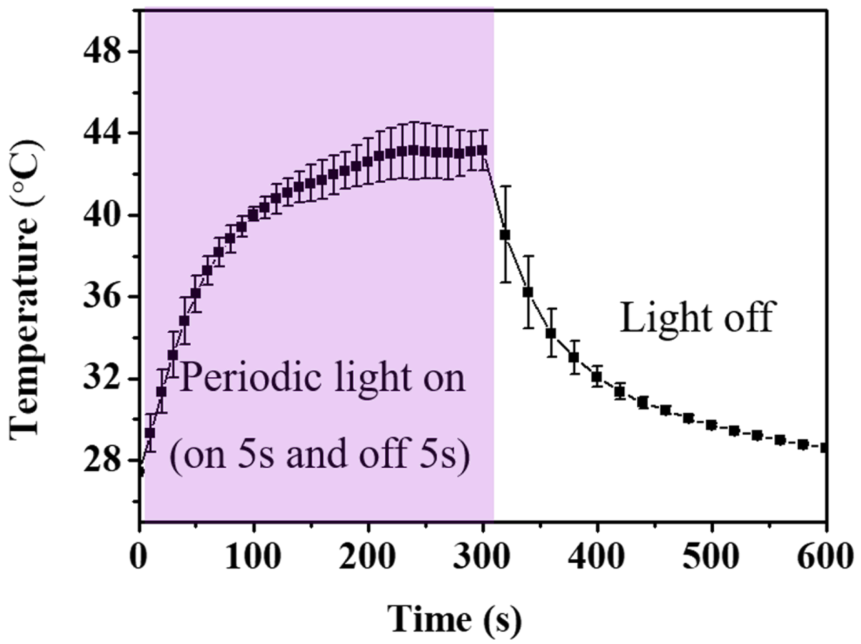

Figure S11. In vitro temperature curve read by temperature sensor during periodic UV-LEDs irradiation (purple area, light on 5s and off 5s for totall 5 min) and after UV-LEDs irradiation.

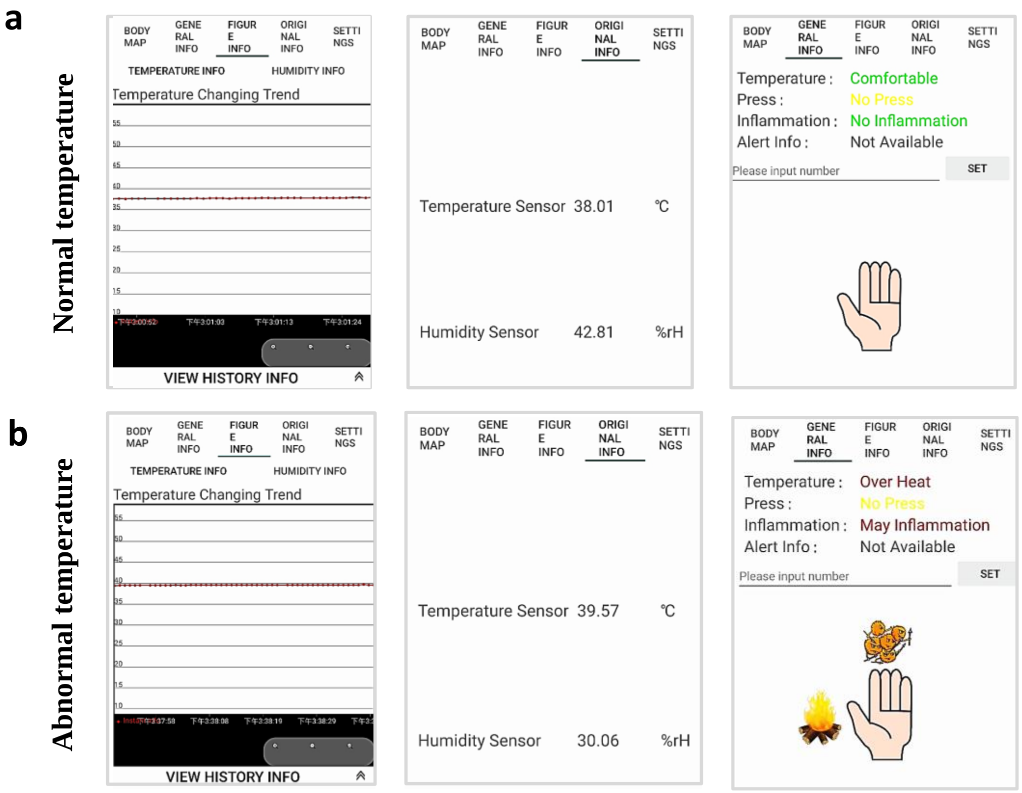

Figure S12. The app interfaces when monitoring normal temperature (a) and abnormal temperature (b).

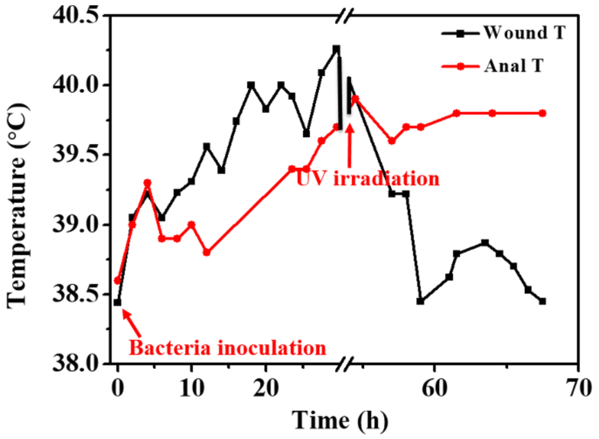

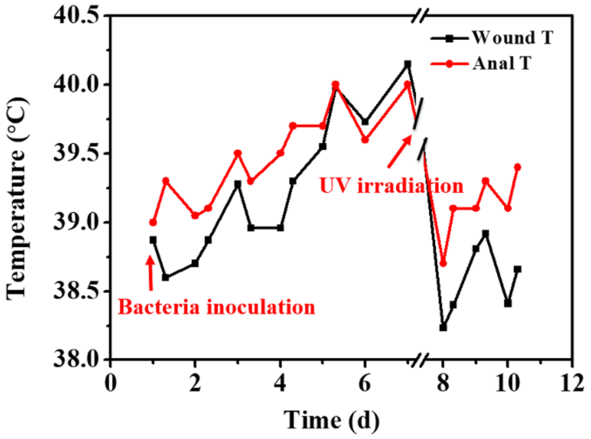

Figure S13. The parallel results of the wound temperature curves recorded by the integrated system in real time. Due to the individual difference of pig on infection susceptibility, the infection-caused temperature increase showed quite different profiles. However, both of them showed the highest values at ~ 40 °C.

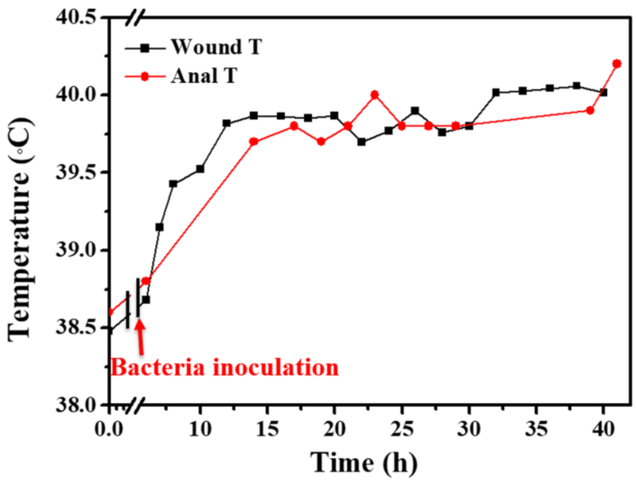

Figure S14. Wound-temperature curve of UR integrated system w/o UV group. Anal temperature was measured as a reference.

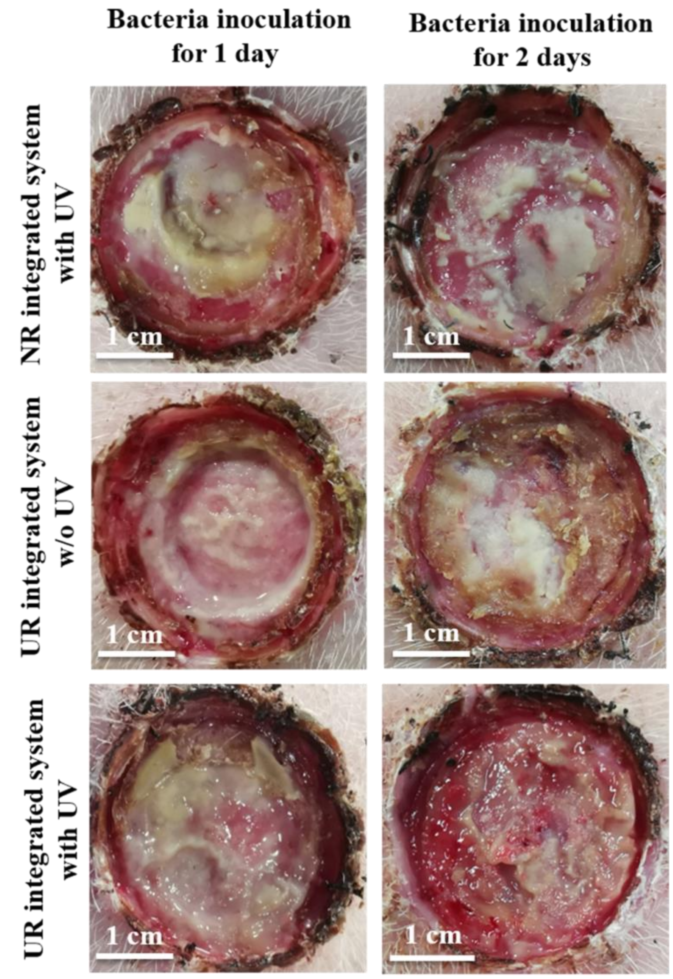

Figure S15. Gross images of the infected wounds treated by different groups after bacteria inoculation for 1 day (left) and 2 days (right) .

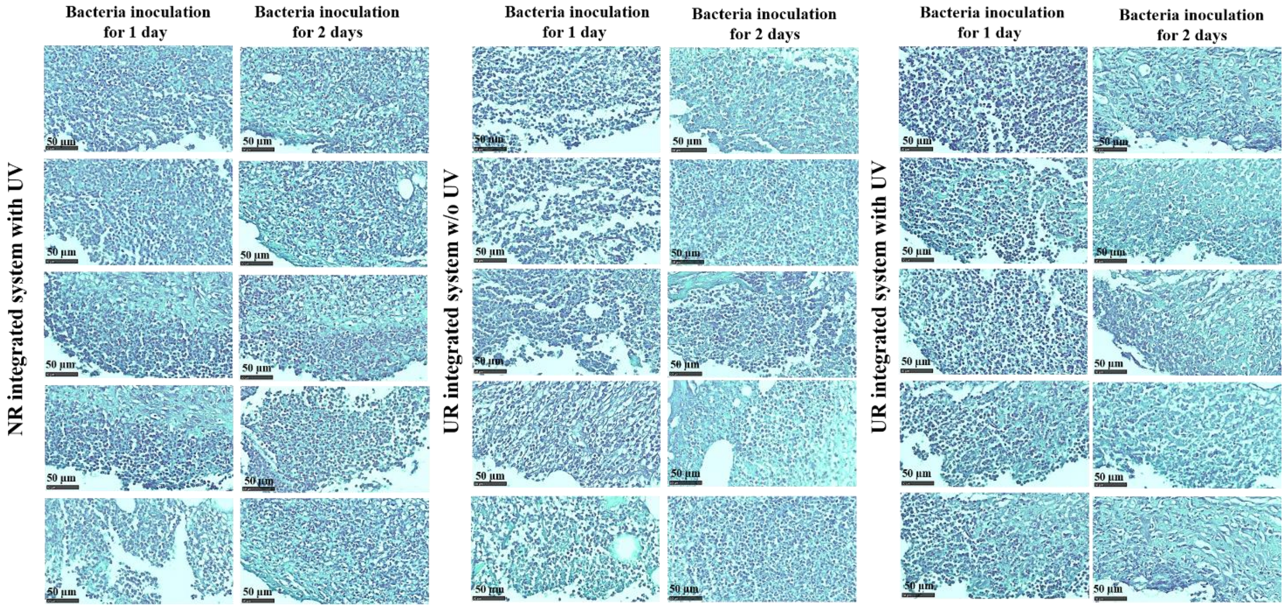

Figure S16. The parallel images of Gram stainings of the wounds treated by different groups after bacteria inoculation for 1 day and 2 days.

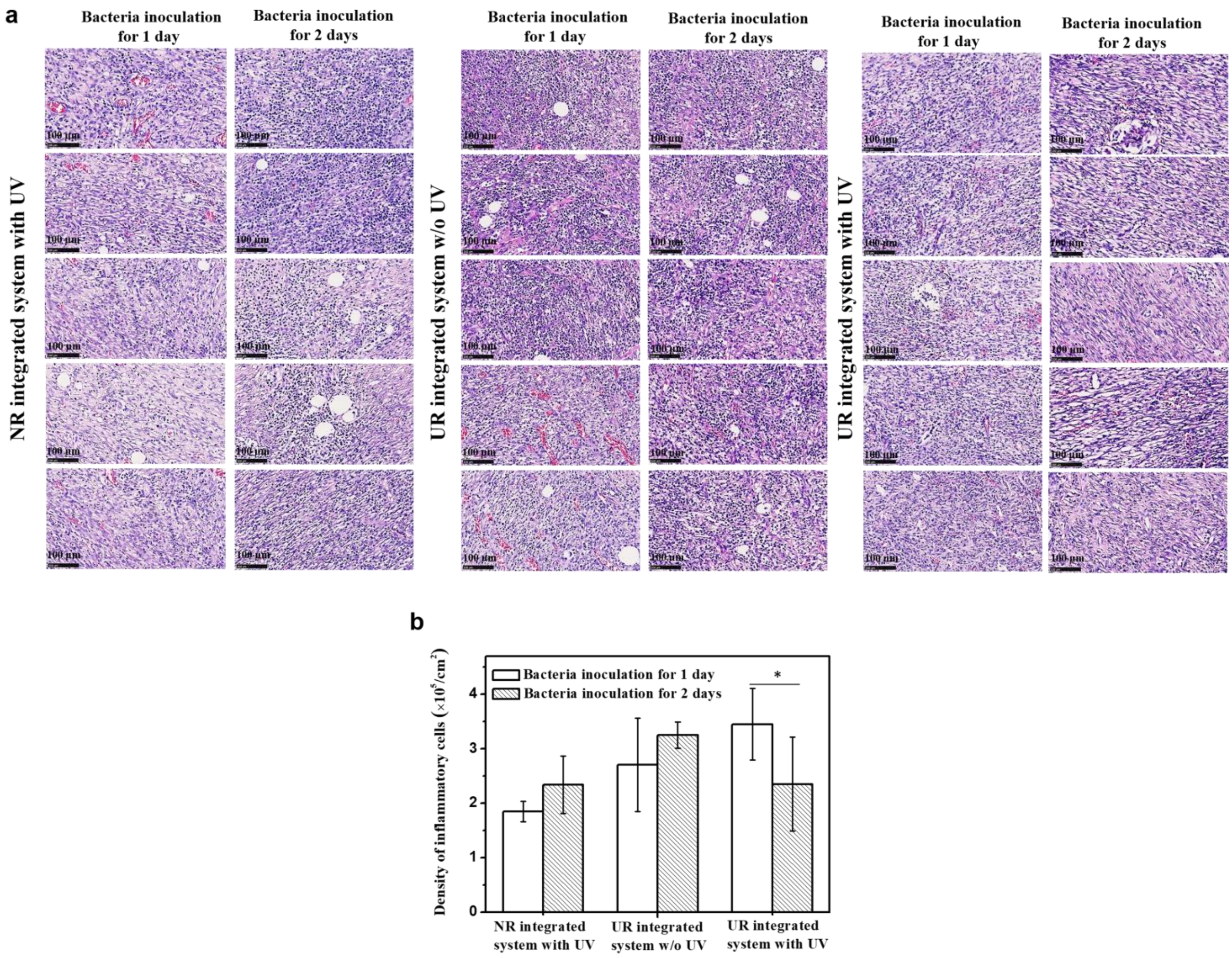

Figure S17. Images (a) and statistical analysis of inflammatory cell densities (b) of HE stainings of the wounds treated by different groups after bacteria inoculation for 1 day and 2 days.

References

[1] S. Kaneko, H. Nakayama, Y. Yoshino, D. Fushimi, K. Yamaguchi, Y. Horiike, J. Nakanishi, *Phys. Chem. Chem. Phys.* **2011**, *13*, 4051.

[2] Q. Pang, X. Zheng, Y. Luo, L. Ma, C. Gao, *J. Mater. Chem. B* **2017**, *5*, 8975.

[3] S. Tang, B. Tian, Q. F. Ke, Z. A. Zhu, Y. P. Guo, *RSC Adv.* **2014**, *4*, 41500.
